# The combination of preoperative platelet count and neutrophil lymphocyte ratio as a prognostic indicator in localized renal cell carcinoma

**DOI:** 10.18632/oncotarget.22688

**Published:** 2017-11-25

**Authors:** Takuya Tsujino, Kazumasa Komura, Atsushi Ichihashi, Takeshi Tsutsumi, Tomohisa Matsunaga, Yuki Yoshikawa, Ryoichi Maenosono, Kyohei Okita, Tomoaki Takai, Rintaro Oide, Koichiro Minami, Hirofumi Uehara, Kohei Taniguchi, Hajime Hirano, Hayahito Nomi, Naokazu Ibuki, Kiyoshi Takahara, Teruo Inamoto, Haruhito Azuma

**Affiliations:** ^1^ Department of Urology, Osaka Medical College, Osaka 569-8686, Japan; ^2^ Translational Research Program, Osaka Medical College, Osaka 569-8686, Japan; ^3^ Department of Biological Fundamental Research, Osaka Medical College, Osaka 569-8686, Japan; ^4^ Department of General and Gastroenterological Surgery, Osaka Medical College, Osaka 569-8686, Japan

**Keywords:** renal cell carcinoma, COP-NLR, NLR, platelet, systemic inflammatory response

## Abstract

**Introduction:**

The combination of platelet count and neutrophil to lymphocyte ratio (COP-NLR) has been shown to provide prognostic information in several cancers, whereas its prognostic value in renal cell carcinoma (RCC) has not been reported. The objective of the present study was to examine the preoperative prognostic value of the COP-NLR in patients with localized RCC undergoing nephrectomy.

**Material and Methods:**

The record of 268 patients, who underwent nephrectomy due to a diagnosis of RCC at our institute was analyzed in the study. The cut-off value of platelet count and NLR were defined by receive operating characteristic (ROC) analysis and the areas under the curve (AUC). Patients with both an increased platelet count (> 310×10^9^/l) and an elevated NLR (> 3.85) were assigned to the score 2, and patients with one or neither of these indicators were assigned to the score 1 or 0, respectively. The impact of the COP-NLR and other clinicopathological characteristics on overall survival (OS) and recurrence-free survival (RFS) were evaluated using the univariate and multivariate Cox regression analysis.

**Result:**

The median follow-up duration after surgical resection was 60 months. Multivariate analysis using the 10 clinicopathological findings selected by univariate analyses demonstrated that the preoperative COP-NLR was an independent prognostic factor for OS (HR: 2.32, 95%CI: 1.22 to 4.26, p=0.011) and RFS (HR: 1.91, 95%CI: 1.02 to 3.53, p=0.044).

**Conclusion:**

The findings of the current study suggested that the preoperative COP-NLR is an independent prognostic indicator of OS and RFS for patients with localized RCC.

## INTRODUCTION

The incidence of renal cell carcinoma (RCC) has increased owing to the aging of population and the advance in imaging technologies [1, 2]. Despite of the progress in the treatment for RCC, nephrectomy is a main-stay as the major treatment option [3], and substantial population (20-30%) of patients who have undergone curative resection of RCC subsequently relapses and deceased [4]. Thus, accurate risk stratification at diagnosis is essential to ensure the best treatment strategy for the patients with RCC.

In the last decade, there have been a number of prognostic factors proposed in the treatment for RCC including biomarkers of inflammation. Increasing evidence supports the involvement of systemic nutritional status and inflammation in cancer progression. Systemic inflammatory response and nutritional decline are closely linked and these conditions have been increasingly recognized as predictive markers. We previously reported that lower body mass index (BMI) and increasing modified Glasgow prognostic score (mGPS), one of the inflammation-based prognostic scores, were associated with poor prognosis [5, 6]. In the last few years, the other inflammation-based prognostic scores, including the neutrophil lymphocyte ratio (NLR), derived neutrophil-to-lymphocyte ratio (dNLR), and platelet lymphocyte ratio (PLR) have been also reported to render prognostic value in many cancers, including RCC [7–11]. In addition, recent studies have demonstrated the combination of platelet count and NLR (COP-NLR) is an independent prognostic factor in colorectal cancer, esophageal squamous cell carcinoma, gastric cancer, hypopharyngeal squamous cell carcinoma and non-small cell lung cancer [12–16]. In the present study, we examined the predictive value of preoperative measurement of COP-NLR for localized RCC patients who underwent curative nephrectomy.

## RESULTS

### Cut-off value of the parameters

**Figure 1:**
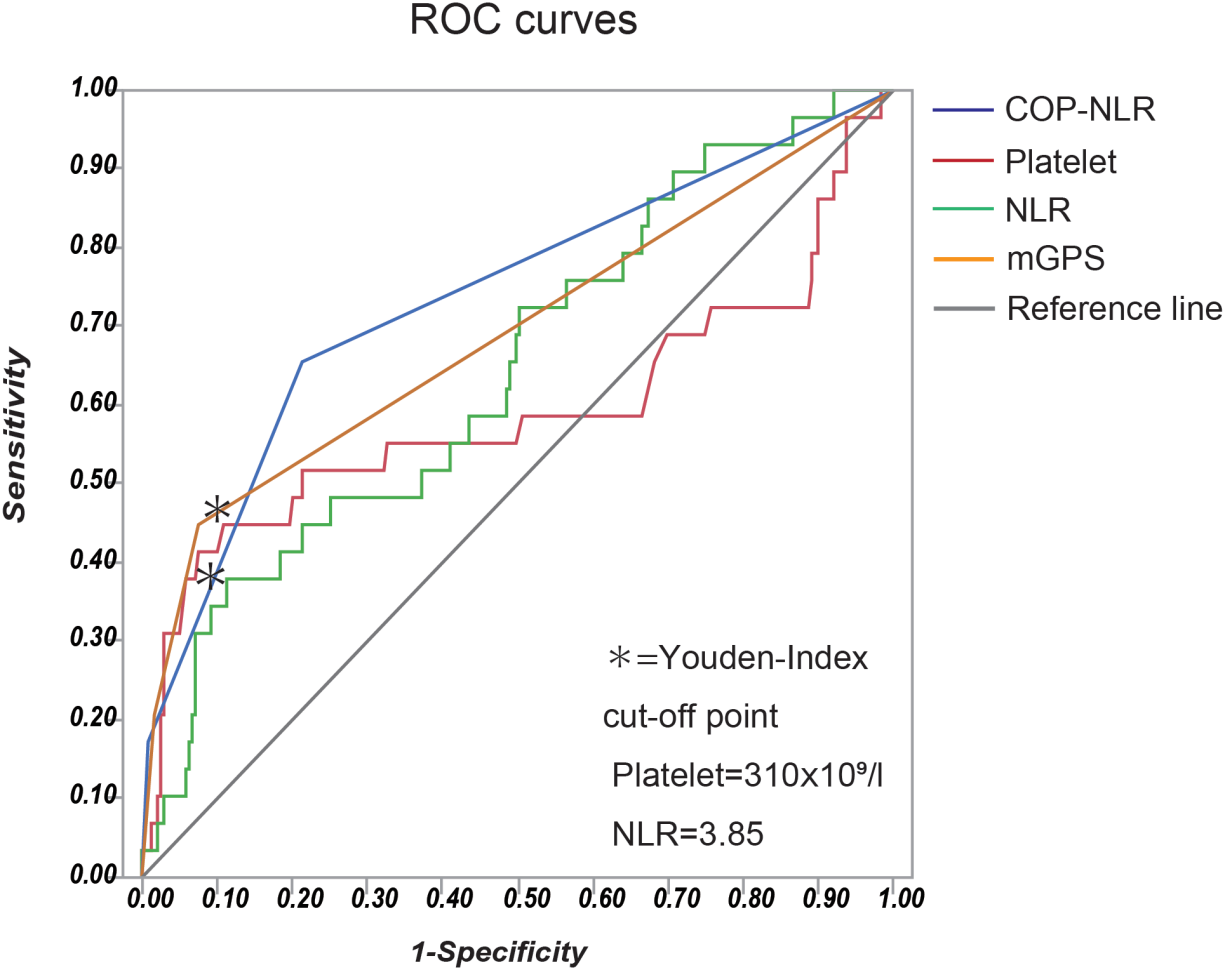
The ROC curves of inflammation-based prognostic scores including the COP-NLR, the NLR, platelet count and the mGPS The Youden-index was applied to determine the optimal cut-off value.

Based on the AUC for survival in the ROC analysis (Figure 1), the Youden index, which maximizes the vertical distance from the reference line, were applied to determine the optimal cut-off values of 3.85 for NLR and 310 × 10^9^ (/l) for platelet count. The COP-NLR in the study was subsequently defined as follows; patients with both elevated platelet count level (> 310 × 10^9^/l) and NLR (> 3.85) were assigned to COP-NLR 2; patients with one of those indicators were assigned to COP-NLR 1; and patients with neither of those were assigned to COP-NLR 0.

### Characteristics in all 268 patients

**Figure 2:**
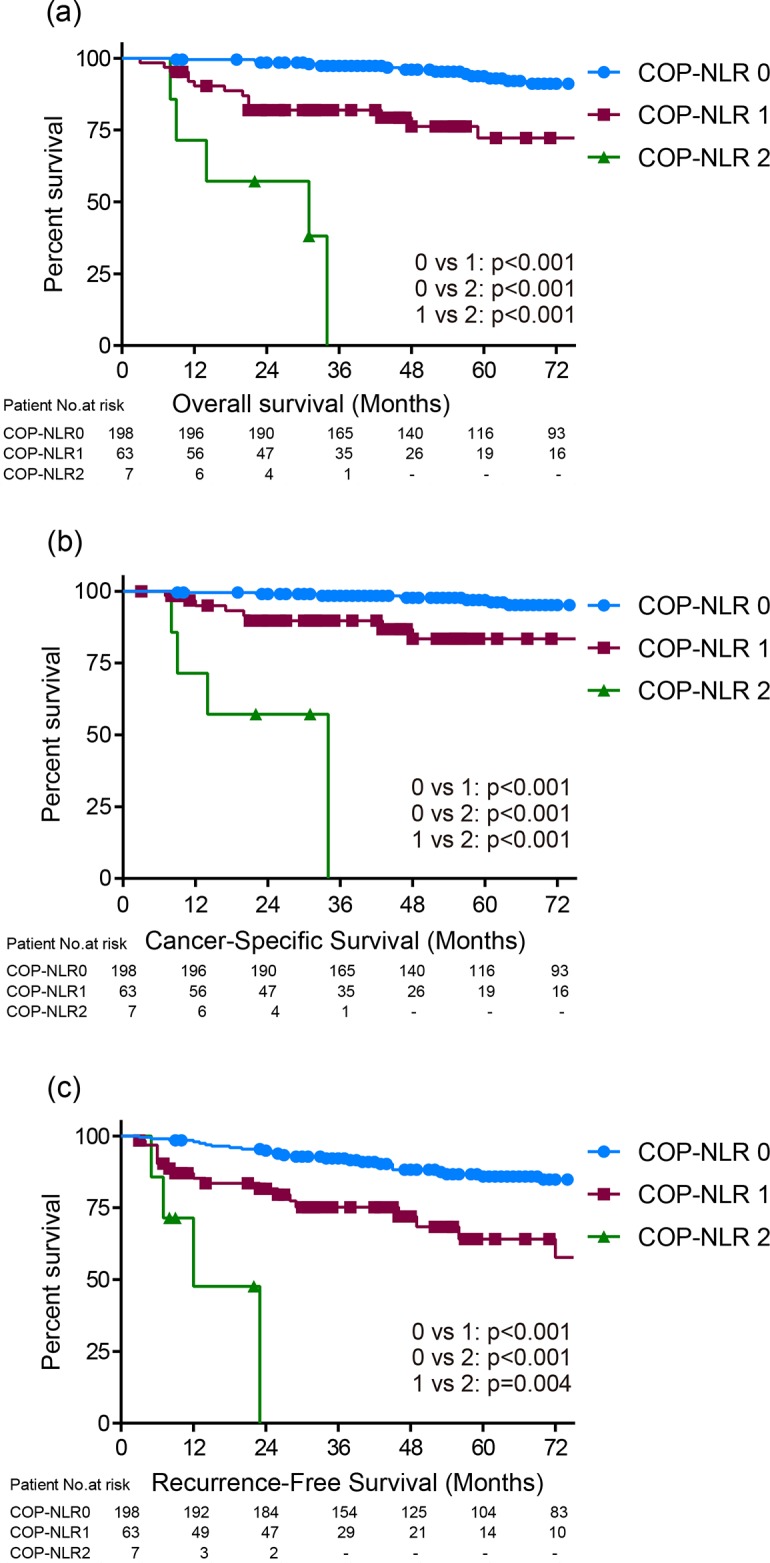
Kaplan-Meier curves of **(a)** overall survival, **(b)** cancer-specific survival, and **(c)** recurrence-free survival according to the COP-NLR (0, 1 and 2) in all 268 patients.

Clinicopathological characteristics in all 268 patients with 60 months of the median follow-up time from surgery are shown in Table 1. A total of 50 (18.6%) patients were died with a median OS of 50 months. Kaplan-Meier estimates showed 87.3% and 92.6% of five-year OS and CSS rates in 268 patients, respectively. There were 198, 63, and 7 patients assigned to the COP-NLR of 0, 1, and 2, respectively. The distribution of characteristics was significantly varied in T classification, tumor size, nuclear grade, tumor necrosis, C-reactive protein, UISS and SSIGN according to COP-NLR. Two and five years OS rates were 98.5 and 93.8% in COP-NLR 0, 82.0 and 72.2% in COP-NLR 1 and 57.1 and 0% in COP-NLR 2, demonstrating a significant difference in their prognosis among the COP-NLR. Kaplan-Meier estimates demonstrated that the higher COP-NLR was significantly associated with shorter OS (log-rank test: p<0.001) (Figure 2a), CSS (2 yrs CSS rate of 99.0% in COP-NLR 0, 89.7% in COP-NLR 1, and 57.1% in COP-NLR 2, log-rank test: p<0.001) (Figure 2b). During follow up, a total of 59 patients relapsed after surgery with median time to recurrence of 25 months. Five years RFS rate in all 268 patients was 80.2%. Kaplan-Meier estimates demonstrated that the increasing COP-NLR was also well-correlated with shorter RFS (2 yrs RFS rate of 94.9% in COP-NLR 0, 81.6% in COP-NLR 1, and 0% in COP-NLR 2, log-rank test: p<0.001) (Figure 2c).

**Table 1:** Baseline characteristics of patients with localized RCC (n=268) according to the COP-NLR

Characteristics	Patients(n=268)	COP-NLR			p value
		0 (n=198)	1 (n=63)	2 (n=7)	
Age (mean±SD)	64.0±11.3	63.8±11.5	64.6±11.3	64.4±6.9	0.871
Age (≤65/>65 years)	126/142	94/104	29/34	4/3	0.956
Sex (male/female)	186/82	141/57	40/23	5/2	0.515
BMI (≤22/>22)	146/122	115/83	23/36	4/3	0.107
ECOG-PS (0/≥1)	222/46	166/32	51/12	5/2	0.653
T classification (I-II/III-IV)	236/32	181/17	50/13	5/2	0.023^*^
Histology type (clear/papillary/chromophobe/others)	241/12/9/6	177/9/8/4	57/3/1/2	7/0/0/0	0.838
Tumor size (cm) (mean±SD)	4.1±2.4	3.9±2.3	4.7±2.5	6.5±2.3	0.002^*^
Tumor size (≤4/>4 cm)	153/115	123/75	29/34	1/6	0.005^*^
Nuclear grade (1-2/3-4)	230/38	177/21	49/14	4/3	0.014^*^
Tumor Necrosis (absent/present)	238/30	182/16	51/12	5/2	0.030^*^
C-reactive protein (mg/l) (mean±SD)	7.5±25.9	2.3±5.7	15.9±35.0	78.2±93.0	<0.001^*^
C-reactive protein (≤2.0/>2.0 mg/l)	193/75	158/40	35/28	0/7	<0.001^*^
UISS (Low/intermediate-high)	164/104	130/68	31/32	3/4	0.042^*^
SSIGN (0-2/≥3)	222/46	171/27	48/15	3/4	0.010^*^
5 year overall survival rate (%)	87.3	93.8	72.2	0	-

COP-NLR: the combination of platelet count and neutrophil lymphocyte ratio, SD: standard deviation, BMI: body mass index, ECOG-PS: eastern cooperative oncology group-performance status, UISS: UCLA integrated staging system, SSIGN: Stage Size Grade Necrosis, ^*^p<0.05.

### Cox regression analysis for OS and RFS

To assess the predictive value for OS, univariate and multivariate analysis were performed (Table 2). Univariate analysis identified several variables significantly associated with OS including BMI (HR: 1.87, 95%CI: 1.06-3.35, p=0.030), ECOG-PS (HR: 2.69, 95%CI: 3.35-14.34, p=0.003), T classification (HR: 6.16, 95%CI: 3.40-10.94, p<0.001), tumor size (HR:3.31, 95%CI: 1.79-6.50, p<0.001), nuclear grade (HR: 3.72, 95%CI: 1.97-6.73, p<0.001), tumor necrosis (HR: 4.12, 95%CI: 2.22-7.41, p<0.001), CRP (HR: 4.00, 95%CI: 2.26-7.10, p<0.001), the UISS (HR: 4.39, 95%CI: 2.39-8.61, p<0.001), the SSIGN (HR: 4.28, 95%CI: 2.42-7.56, p<0.001) and the COP-NLR (HR: 4.59, 95%CI: 2.59-8.14, p<0.001). On multivariate analysis adjusting for those variables exhibited significant associations in univariate analysis, four variables including T classification (HR: 3.56, 95%CI: 1.46-8.71, p=0.005), tumor necrosis (HR: 2.93, 95%CI: 1.21-7.25, p=0.017), CRP (HR: 2.63, 95%CI: 1.30-5.27, p=0.008) and the COP-NLR (HR: 2.32, 95%CI: 1.22-4.36, p=0.011) still remained as significant predictors for OS.

**Table 2:** Univariate and multivariate analysis for OS in patients who had no metastasis at the time of nephrectomy (n=268)

Characteristics	OS			
	Univariate		Multivariate	
	HR (95% CI)	p value	HR (95% CI)	p value
Age (≤65/>65 years)	1.43 (0.79-2.58)	0.232		
BMI (≤22/>22)	1.87 (1.06-3.35)	0.030^*^	1.71 (0.90-3.28)	0.099
Sex (male/female)	0.91 (0.46-1.69)	0.773	-	-
ECOG-PS (0/≥1)	2.69 (1.43-4.85)	0.003^*^	1.44 (0.65-3.13)	0.366
T classification (I-II/III-IV)	6.16 (3.40-10.94)	<0.001^*^	3.56 (1.46-8.71)	0.005^*^
Histology type (clear/others)	0.87 (2.62-2.16)	0.792		
Tumor size (cm) (≤4.0/>4.0)	3.31 (1.79-6.50)	<0.001^*^	1.63 (0.73-3.69)	0.230
Nuclear grade (1-2/3-4)	3.72 (1.97-6.73)	<0.001^*^	1.02 (0.44-2.29)	0.971
Tumor Necrosis (absent/present)	4.12 (2.22-7.41)	<0.001^*^	2.93 (1.21-7.25)	0.017^*^
C-reactive protein (mg/l) (≤2.0/>2.0)	4.00 (2.26-7.10)	<0.001^*^	2.63 (1.30-5.27)	0.008^*^
UISS (Low/intermediate-high)	4.39 (2.39-8.61)	<0.001^*^	1.61 (0.66-3.93)	0.293
SSIGN (0-2/≥3)	4.28 (2.42-7.56)	<0.001^*^	0.40 (0.13-1.27)	0.123
COP-NLR (0/1-2)	4.59 (2.59-8.14)	<0.001^*^	2.32 (1.22-4.36)	0.011^*^

OS: overall survival, HR: hazard ratio, CI: confidence interval, BMI: body mass index, ECOG-PS: eastern cooperative oncology group-performance status, UISS: UCLA integrated staging system, SSIGN: Stage Size Grade Necrosis, COP-NLR: the combination of platelet count and neutrophil lymphocyte ratio, ^*^p<0.05.

Since the present study was originally designated to the patients who had no metastasis at the time of surgery, we then assessed the predictive value for RFS (Table 3). Univariate analysis identified several variables significantly associated with RFS including T classification (HR: 7.22, 95%CI: 4.00-12.72, p<0.001), tumor size (HR:4.00, 95%CI: 2.23-7.64, p<0.001), nuclear grade (HR: 4.25, 95%CI: 2.35-7.44, p<0.001), tumor necrosis (HR: 2.79, 95%CI: 1.42-5.11, p=0.004), CRP (HR: 4.32, 95%CI: 2.50-7.55, p<0.001), the UISS (HR: 3.17, 95%CI: 1.83-5.64, p<0.001), the SSIGN (HR: 4.09, 95%CI: 2.34-7.05, p<0.001) and the COP-NLR (HR: 3.70, 95%CI: 2.10-6.46, p<0.001). On multivariate analysis adjusting for those variables exhibited significant associations in univariate analysis, three variables including T classification (HR: 3.72, 95%CI: 1.60-8.90, p=0.002), CRP (HR: 2.52, 95%CI: 1.37-4.63, p=0.003) and the COP-NLR (HR: 1.91, 95%CI: 1.02-3.53, p=0.044) still remained as significant predictors for RFS.

**Table 3:** Univariate and multivariate analysis for RFS in patients who had no metastasis at the time of nephrectomy (n=268)

Characteristics	RFS			
	Univariate		Multivariate	
	HR (95% CI)	p value	HR (95% CI)	p value
Age (≤65/>65 years)	1.62 (0.90-3.09)	0.111	-	-
BMI (≤22/>22)	1.49 (0.87-2.57)	0.149	-	-
Sex (male/female)	1.11 (0.61-1.95)	0.717	-	-
ECOG-PS (0/≥1)	1.66 (0.83-3.08)	0.141	-	-
T classification (I-II/III-IV)	7.22 (4.00-12.72)	<0.001^*^	3.72 (1.60-8.90)	0.002^*^
Histology type (clear/others)	0.73 (0.22-1.80)	0.536	-	-
Tumor size (cm) (≤4.0/>4.0)	4.00 (2.23-7.64)	<0.001^*^	2.04 (0.99-4.26)	0.052
Nuclear grade (1-2/3-4)	4.25 (2.35-7.44)	<0.001^*^	1.79 (0.86-3.79)	0.120
Tumor Necrosis (absent/present)	2.79 (1.42-5.11)	0.004^*^	1.17 (0.49-2.83)	0.713
C-reactive protein (mg/l) (≤2.0/>2.0)	4.32 (2.50-7.55)	<0.001^*^	2.52 (1.37-4.63)	0.003^*^
UISS (Low/intermediate-high)	3.17 (1.83-5.64)	<0.001^*^	1.01 (0.45-2.18)	0.982
SSIGN (0-2/≥3)	4.09 (2.34-7.05)	<0.001^*^	0.72 (0.26-1.99)	0.531
COP-NLR (0/1-2)	3.70 (2.10-6.46)	<0.001^*^	1.91 (1.02-3.53)	0.044^*^

RFS: recurrence-free survival, HR: hazard ratio, CI: confidence interval, BMI: body mass index, ECOG-PS: eastern cooperative oncology group-performance status, UISS: UCLA integrated staging system, SSIGN: Stage Size Grade Necrosis, COP-NLR: the combination of platelet count and neutrophil lymphocyte ratio, ^*^p<0.05.

### Predicting value of COP-NLR comparing to the other variables

As previous studies have shown the predictive value of individual inflammatory indicators [11, 17], we examined the impact of the NLR and platelet count on OS and RFS in the present cohort. On multivariate analysis, the NLR and platelet count were independent prognostic factors (Tables 4 and 5). Subsequently, we compared ROC curves in those variables to assess the clinical implications including the COP-NLR. As shown in Figure 1 and Table 6, the AUC values of the COP-NLR were 0.79 (3 years) and 0.74 (5 years) for the prediction on OS, which were comparable to those of the NLR (3 years: AUC=0.72, p=0.187; 5 years: AUC=0.65, p=0.041), platelet count (3 years: AUC=0.65, p=0.042; 5 years: AUC=0.59, p=0.016) and the mGPS (3 years: AUC=0.73, p=0.258; 5 years: AUC=0.69, p=0.319). These data imply the utility of COP-NLR as a significant predicting indicator compared to other variables.

**Table 4:** Univariate and multivariate analysis for OS in patients who had no metastasis at the time of nephrectomy (n=268)

Characteristics	OS			
	Univariate		Multivariate	
	HR (95% CI)	p value	HR (95% CI)	p value
Age (≤65/>65 years)	1.43 (0.79-2.58)	0.232		
BMI (≤22/>22)	1.87 (1.06-3.35)	0.030^*^	1.70 (0.89-3.29)	0.105
Sex (male/female)	0.91 (0.46-1.69)	0.773	-	-
ECOG-PS (0/≥1)	2.69 (1.43-4.85)	0.003^*^	1.35 (0.57-3.20)	0.489
T classification (I-II/III-IV)	6.16 (3.40-10.94)	<0.001^*^	3.50 (1.45-8.56)	0.005^*^
Histology type (clear/others)	0.87 (2.62-2.16)	0.792		
Tumor size (cm) (≤4.0/>4.0)	3.31 (1.79-6.50)	<0.001^*^	1.58 (0.70-3.56)	0.266
Nuclear grade (1-2/3-4)	3.72 (1.97-6.73)	<0.001^*^	0.97 (0.43-2.23)	0.958
Tumor Necrosis (absent/present)	4.12 (2.22-7.41)	<0.001^*^	2.89 (1.18-7.26)	0.020^*^
C-reactive protein (mg/l) (≤2.0/>2.0)	4.00 (2.26-7.10)	<0.001^*^	2.51 (1.22-5.09)	0.013^*^
UISS (Low/intermediate-high)	4.39 (2.39-8.61)	<0.001^*^	1.68 (0.67-4.17)	0.266
SSIGN (0-2/≥3)	4.28 (2.42-7.56)	<0.001^*^	0.39 (0.12-1.23)	0.109
Platelet count (10^9^/l) (≤310/>310)	4.57 (2.45-8.23)	<0.001^*^	2.69 (1.29-5.45)	0.009^*^
NLR (≤3.85/>3.85)	3.82 (1.93-7.11)	<0.001^*^	2.65 (1.23-5.46)	0.014^*^

OS: overall survival, HR: hazard ratio, CI: confidence interval, BMI: body mass index, ECOG-PS: eastern cooperative oncology group-performance status, UISS: UCLA integrated staging system, SSIGN: Stage Size Grade Necrosis, NLR: neutrophil lymphocyte ratio, ^*^p<0.05.

**Table 5:** Univariate and multivariate analysis for RFS in patients who had no metastasis at the time of nephrectomy (n=268)

Characteristics	RFS			
	Univariate		Multivariate	
	HR (95% CI)	p value	HR (95% CI)	p value
Age (≤65/>65 years)	1.62 (0.90-3.09)	0.111	-	-
BMI (≤22/>22)	1.49 (0.87-2.57)	0.149	-	-
Sex (male/female)	1.11 (0.61-1.95)	0.717	-	-
ECOG-PS (0/≥1)	1.66 (0.83-3.08)	0.141	-	-
T classification (I-II/III-IV)	7.22 (4.00-12.72)	<0.001^*^	3.64 (1.66-8.20)	0.001^*^
Histology type (clear/others)	0.73 (0.22-1.80)	0.536	-	-
Tumor size (cm) (≤4.0/>4.0)	4.00 (2.23-7.64)	<0.001^*^	2.22 (1.14-4.45)	0.019^*^
Nuclear grade (1-2/3-4)	4.25 (2.35-7.44)	<0.001^*^	1.75 (0.87-3.60)	0.120
Tumor Necrosis (absent/present)	2.79 (1.42-5.11)	0.004^*^	1.36 (0.60-3.15)	0.460
C-reactive protein (mg/l) (≤2.0/>2.0)	4.32 (2.50-7.55)	<0.001^*^	2.19 (1.20-3.98)	0.011^*^
UISS (Low/intermediate-high)	3.17 (1.83-5.64)	<0.001^*^	1.15 (0.54-2.36)	0.703
SSIGN (0-2/≥3)	4.09 (2.34-7.05)	<0.001^*^	0.58 (0.22-1.49)	0.263
Platelet count (10^9^/l) (≤310/>310)	3.76 (2.07-6.55)	<0.001^*^	2.08 (1.08-3.84)	0.029^*^
NLR (≤3.85/>3.85)	4.49 (2.46-7.87)	<0.001^*^	3.67 (1.91-6.77)	<0.001^*^

RFS: recurrence-free survival, HR: hazard ratio, CI: confidence interval, BMI: body mass index, ECOG-PS: eastern cooperative oncology group-performance status, UISS: UCLA integrated staging system, SSIGN: Stage Size Grade Necrosis, NLR: neutrophil lymphocyte ratio, ^*^p<0.05.

**Table 6:** Comparison of the discriminatory ability for the prediction on OS in COP-NLR, NLR, platelet count and mGPS

Period	AUC	95%CI	p value
**3 years**			
COP-NLR	0.79	0.67-0.88	
NLR	0.72	0.60-0.82	0.187
Platelet count	0.65	0.48-0.79	0.042^*^
mGPS	0.73	0.60-0.83	0.258
**5 years**			
COP-NLR	0.74	0.63-0.82	
NLR	0.65	0.53-0.75	0.041^*^
Platelet count	0.59	0.42-0.72	0.016^*^
mGPS	0.69	0.59-0.78	0.319

AUC: area under the curve, CI: Confidence interval, OS: overall survival, COP-NLR: the combination of platelet count and neutrophil lymphocyte ratio, NLR: neutrophil count to lymphocyte count ratio, mGPS: modified Glasgow Prognostic Score, ^*^p<0.05.

### Association between major treatment options

**Figure 3:**
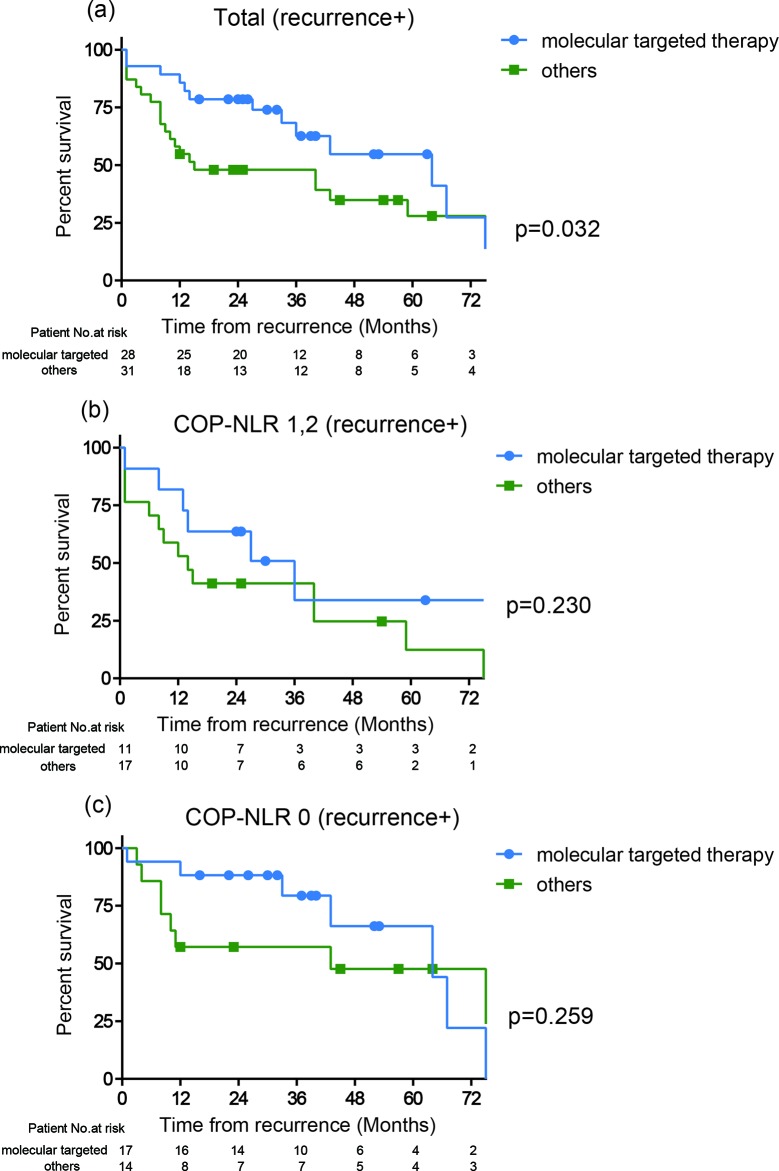
Kaplan-Meier curves of survival from the time of recurrence according to the treatment after recurrence (molecular targeted or others) in patient who relapse after surgery with **(a)** all of the COP-NLR, **(b)** the COP-NLR 0, and **(c)** the higher COP-NLR.

Since molecular targeted drugs are considered as a significant factor, which impacts the treatment outcomes in RCC, we assessed the distribution of the patients who were treated with molecular targeted drugs according to the COP-NLR. There were 28 patients (17 patients in COP-NLR 0, 10 patients in COP-NLR 1 and 1 patient in COP-NLR 2, respectively), who had molecular targeted therapy after recurrence, while one patient had the drug as neoadjuvant therapy as well. There was no patient who had the drug as adjuvant therapy. In the total cohort, 59 patients relapsed postoperatively, 36 patients (61%) of whom deceased during follow up. Clinicopathological characteristics in 59 patients who relapsed after surgery are shown in Table 7. There were 31, 22, and 6 patients assigned to the COP-NLR of 0, 1, and 2, respectively, and no significant difference between the COP-NLR and molecular target therapies was seen (p=0.199). Since all of cases with molecular targeted therapies were administrated after patients relapsed following to nephrectomy, we therefore assessed whether treatment outcome of the therapy differs between the COP-NLR from the time point of recurrence. As expected, the patients with the therapies (28 patients) had significantly favorable OS compared to those without therapy (31 patients) (HR: 2.10, 95%CI: 1.05–4.37, p=0.033) from the time point of recurrence (Figure 3a). We then stratified those 59 patients into COP-NLR 0 (31 patients) and COP-NLR 1, 2 (28 patients) to assess whether the COP-NLR could predict the treatment outcome of those agents at the time point of recurrence. Kaplan-Meier estimates demonstrated that both of the groups showed the trend of better outcome in patients with molecular targeted therapies despite of not achieving statistical significance (Figure 3b, 3c). In the patients with COP-NLR 0, the median OS from the recurrence were 64 and 43 months in patients with and without molecular targeted therapies, respectively (HR: 1.81, 95%CI: 0.65–5.46, p=0.259). In the patients with higher (1 and 2) COP-NLR, the median OS from the recurrence were 36 and 14 months in patients with and without molecular targeted therapies, respectively (HR: 1.77, 95%CI: 0.71–5.02, p=0.230). Although the small cohort size after the stratification, there seemed to be no correlation between the effect of molecular targeted drugs and COP-NLR.

**Table 7:** Baseline characteristics of patients who relapsed after surgery according to the COP-NLR (n=59)

Characteristics	Patients(n=59)	COP-NLR			p value
		0 (n=31)	1 (n=22)	2 (n=6)	
Age (mean±SD)	64.0±11.3	66.3±9.2	64.4±9.9	63.8±7.4	0.707
Sex (male/female)	42/17	22/9	16/6	4/2	0.959
BMI (≤22/>22)	27/32	16/15	7/15	4/2	0.196
ECOG-PS (0/≥1)	45/14	26/5	15/7	4/2	0.349
T classification (I-II/III-IV)	38/21	21/10	13/9	4/2	0.806
Histology type (clear/papillary/chromophobe/others)	55/1/2/1	28/1/2/0	21/0/0/1	6/0/0/0	0.434
Tumor size (cm) (mean±SD)	4.1±2.4	5.2±2.3	6.0±2.6	6.9±2.2	0.238
Nuclear grade (1-2/3-4)	40/19	24/7	12/10	4/2	0.215
Tumor Necrosis (absent/present)	44/15	26/5	14/8	4/2	0.220
C-reactive protein (mg/l) (mean±SD)	7.5±25.9	3.8±7.2	34.4±52.3	85.7±99.5	<0.001^*^
UISS (Low/intermediate-high)	22/37	12/19	7/15	3/3	0.700
SSIGN (0-2/≥3)	35/24	20/11	12/10	3/3	0.680
Treatment after recurrence (molecular targeted therapy/others)	28/31	17/14	10/12	1/5	0.199

COP-NLR: the combination of platelet count and neutrophil lymphocyte ratio, SD: standard deviation, BMI: body mass index, ECOG-PS: eastern cooperative oncology group-performance status, UISS: UCLA integrated staging system, SSIGN: Stage Size Grade Necrosis, ^*^p<0.05.

Next, we assessed the association of prognosis between operative procedures, namely partial or radical nephrectomy (PN or RN) to examine the hypothesis that patients with RCC harboring high grade or small pT3 who underwent PN might be allocated to the higher COP-NLR and had poorer prognosis. In the total cohort, 40 patients underwent PN. Clinicopathological characteristics of those patients according to the COP-NLR are shown in Table 8. Of them, there was actually no patient with T3 or high grade. However, of those 40 patients who underwent PN, higher COP-NLR had significantly poorer prognosis than the COP-NLR 0. These data indicated the poorer prognosis in higher COP-NLR for the patients who had PN and drove us to the next hypothesis that those patients might have benefited from canonical radical nephrectomy. Accordingly, we assessed the correlation between operative procedure and clinical outcome in patients with the higher COP-NLR (1 and 2). Clinicopathological characteristics of those patients according to operative procedure are shown in Table 9. There were 65 patients who underwent radical nephrectomy (RN), while only 5 patients underwent PN in patients with higher COP-NLR. The distribution of characteristics significantly varied only in tumor size. Kaplan-Meier estimates demonstrated that there was no significant difference on OS and RFS between PN and RN for those patient with higher COP-NLR (data not shown).

**Table 8:** Baseline clinicopathological findings in 40 patients who underwent partial nephrectomy according to the COP-NLR

Characteristics	Patients(n=40)	COP-NLR		
		0 (n=35)	1, 2 (n=5)	p value
Age (mean±SD)	62.9±14.4	63.2±14.5	61.0±15.1	0.757
Sex (male/female)	28/12	25/10	3/2	0.610
BMI (≤22/>22)	23/17	20/15	3/2	0.904
ECOG-PS (0/≥1)	37/3	33/2	4/1	0.324
T classification (I/II/III/IV)	40/0/0/0	35/0/0/0	5/0/0/0	1.000
Histology type (clear/papillary/chromophobe/others)	34/4/2/0	29/4/2/0	5/0/0/0	0.418
Tumor size (cm) (mean±SD)	2.1±0.9	2.2±0.2	1.8±0.4	0.464
Nuclear grade (1-2/3-4)	37/3	32/3	5/0	0.361
Tumor Necrosis (absent/present)	38/2	33/2	5/0	0.459
C-reactive protein (mg/l) (mean±SD)	1.8±3.4	1.4±2.4	4.6±7.3	0.042^*^
5 year overall survival rate (%)	93.1	95.4	80	-

COP-NLR: the combination of platelet count and neutrophil lymphocyte ratio, SD: standard deviation, BMI: body mass index, ECOG-PS: eastern cooperative oncology group-performance status, ^*^p<0.05.

**Table 9:** Baseline clinicopathological findings of patients with the higher COP-NLR (1, 2) according to operative procedure

Characteristics	Patients(n=70)	operative procedure		
		radical (n=65)	partial (n=5)	p value
Age (mean±SD)	64.6±10.9	64.2±10.6	61.0±15.1	0.450
Sex (male/female)	45/25	42/23	3/2	0.837
BMI (≤22/>22)	31/39	28/37	3/2	0.464
ECOG-PS (0/≥1)	56/14	52/13	4/1	1.000
T classification (I/II/III/IV)	48/7/13/2	43/7/13/2	5/0/0/0	0.267
Histology type (clear/papillary/chromophobe/others)	64/3/1/2	59/3/1/2	5/0/0/0	0.818
Tumor size (cm) (mean±SD)	4.1±2.4	4.5±2.4	1.8±0.4	<0.001^*^
Nuclear grade (1-2/3-4)	53/17	48/17	5/0	0.088
Tumor Necrosis (absent/present)	56/14	51/14	5/0	0.127
C-reactive protein (mg/l) (mean±SD)	22.1±47.0	23.5±48.5	4.6±7.3	0.391
5 year overall survival rate (%)	66.6	66.1	80.0	-

COP-NLR: the combination of platelet count and neutrophil lymphocyte ratio, SD: standard deviation, BMI: body mass index, ECOG-PS: eastern cooperative oncology group-performance status, ^*^p<0.05.

## DISCUSSION

In the present study, we assessed the prognostic value of preoperative assessment using COP-NLR in patients with localized RCC, who underwent partial or radical nephrectomy, and showed that the increasing COP-NLR was significantly associated with shorter OS and RFS. The results demonstrated that COP-NLR is an independent prognostic factor for patients with RCC after nephrectomy. By now, several studies have shown a relationship between the COP-NLR and prognosis in patients with various types of cancers [12–16] suggesting its prognostic value, and the current study is, to our knowledge, the first study to assess the prognostic value of preoperative COP-NLR in patients with RCC.

For the last decades, an association between preoperative systemic inflammatory response and a poorer postoperative survival has been reported. Accumulated evidence has demonstrated that the systemic inflammatory biomarkers including NLR, dNLR, PLR, CRP, GPS and mGPS represent independent prognostic factors for various types of cancer including RCC [6, 7, 11, 18, 19]. In addition, the present study indicated that elevated COP-NLR was significantly associated with poor prognosis in RCC patients, who underwent curative nephrectomy. Recent studies have demonstrated that systemic inflammatory response was associated with reactive thrombocytosis in several types of cancer including RCC [20–22]. Thrombocytosis generally occurs in 10–57% of patients with cancer [23, 24]. Reactive thrombocytosis is induced in a background of hypercytokinemia, and Interleukin-6 (IL-6) has an important role in reactive thrombocytosis [25], stimulating elevated CRP level in the liver [26]. IL-6 has been reported to induce not only neutrophil proliferation but also the differentiation of megakaryocytes to platelets [27, 28], leading to the aberrant systemic inflammatory response. In addition, thrombocytosis is also induced by the tumor itself [29]. Several studies have revealed that vascular endothelial growth factor (VEGF) stimulates megakaryocyte differentiation [30]. Given the role of VEGF in tumor growth, quantitative evaluation of thrombocytosis might indirectly reflect tumor progression serving as a surrogate marker of tumor burden [31]. As shown in Table 6, we compared AUC for predicting OS in those systemic inflammatory biomarkers including mGPS, NLR, platelet count, and COP-NLR. Although there was no significant difference between those variables, COP-NLR exhibited the highest value of AUC implying this combination use of NLR and platelet count as a reliable surrogate marker predicting OS for RCC.

In the present study, we applied two widely validated prognostic scoring models including UISS and SSIGN to compare the predictive value of COP-NLR on multivariate analysis. The UISS score was proposed in UCLA, and the score is assigned by tumor stage, Fuhrman grade and ECOG-PS [32]. The SSIGN score was proposed in Mayo clinic, and the score is assigned based on T stage, nodal disease, tumor size, nuclear grade, presence or absence of tumor necrosis, and the presence or absence of metastases [33]. Those scoring models both include perioperative and pathological findings such as Fuhrman grade, presence of necrosis, and ECOG-PS. The COP-NLR, on the other hand, consists of platelet count and calculation of NLR, which is routinely collected in preoperative blood draw. Therefore, the COP-NLR might render an objective premise prior to the treatment in patients with operable RCC, which potentially allows physicians to consider targeted neoadjuvant and adjuvant therapy in patients with higher COP-NLR.

In the present cohort, the proportion of COP-NLR 2 was small (3%), whereas those patients ultimately had worse prognosis (5 year OS rate of 0%). Kaplan-Meier estimates illustrated significant differences among COP-NLR on OS (2 yrs survival rate of 98.5 in COP-NLR 0, 82.0% in COP-NLR 1 and 57.1% in COP-NLR 2), and RFS (2 yrs RFS rate of 94.9% in COP-NLR 0, 81.6% in COP-NLR 1 and 47.6% in COP-NLR 2). Multivariate analysis revealed that COP-NLR of ≥ 1 was an independent predictor for the lethality and recurrent progression beyond the other major factors including UISS, SSIGN, and ECOG-PS. These data suggest that patients with the COP-NLR of ≥ 1 might be considered multimodal therapeutic approach in addition to conventional curative nephrectomy. The COP-NLR has the advantage of identifying these patients preoperatively, and potentially offer the chance of novel neoadjuvant therapies without decline of renal function by nephrectomy. Given that neoadjuvant therapy using approved molecular targeted agents are still controversial [34–37], preoperative assessment using the COP-NLR might offer the valid information for identifying the patients who are more likely to benefit from those therapies prior to nephrectomy.

The limitations of our study include its retrospective, single-institution design and the small sample size, which precluded to determine whether COP-NLR offers the prediction of treatment outcome for molecular targeted drugs for the patients after relapse and better decision making for operation (i.e. PN and RN). In addition, other putative patient statuses, such as diabetes mellitus, hypertension, cardiovascular disease, smoking etc [38–40], which have been shown to be prognostic factors for RCC patients, were not examined in the current study. Larger prospective randomized controlled trials are needed to confirm our preliminary findings.

## MATERIALS AND METHODS

### Patients

The subjects of the retrospective study was a cohort of 313 consecutive RCC patients who underwent nephrectomy at Osaka Medical College Hospital from 2002 to 2015. Patients with metastasis at the time of nephrectomy or those who had any missing clinicopathological information were excluded from the study. Accordingly, 268 patients were included in the analysis. The study was designated in accordance with the ethical standards of the World Medical Association Declaration of Helsinki [41]. Written informed consent was obtained from all participants for the study registry.

### Clinico-pathological characteristics

Clinical stage examined by computer tomography (CT), magnetic resonance imaging (MRI), ultrasound, and chest-X ray, and other patient information including performance status (Eastern Cooperative Oncology Group, ECOG-PS) were preoperatively recorded. Pathological review including Fuhrman nuclear grade [42] was examined in all patients as well as the 7th TNM classification of the UICC and AJCC guidelines of renal tumors. Routine laboratory measurements including platelet count, neutrophil count, lymphocyte count, and C-reactive protein level were determined preoperatively (one to two weeks before the surgery). The University of California, Los Angeles (UCLA) Integrated Staging System (UISS) score was assigned as previously described [32], stratifying patients into low, intermediate or high risk groups according to the combination of tumor stage, Fuhrman grade, and ECOG-PS. The Stage Size Grade Necrosis (SSIGN) score was derived as previously described [33]. In short, patients were awarded scores based on T stage, nodal disease, tumor size, nuclear grade, presence or absence of tumor necrosis, and the presence or absence of metastases, followed by classification into five stages (0–2/3,4/5,6/7–9/>10) according to the score.

### Follow-up

Follow-up schedules were applied referring to the NCCN Clinical Practice Guidelines. Follow up CT and Chest X-ray were examined to detect any findings suspected to disease progression every three months in the first year. Thereafter, patients were followed up every six months. Follow-up was calculated from the day of surgery to the day of death or the last visit.

### Statistical analysis

Overall survival (OS) was calculated from the date of surgery to the date of death or last follow-up. Cancer-specific survival (CSS) was calculated from the date of surgery to the date of cancer death or last follow-up. Recurrence-free survival (RFS) was calculated from the date of surgery to the date of disease recurrence or metastasis or the last follow-up. The optimal cut-off points for the inflammation-based factors including platelet count, NLR, and other inflammation indicators were determined by receive operating characteristic (ROC) analysis and the areas under the curve (AUC) were calculated and compared as previously described [43]. Clinicopathological findings in the analysis included patient age, sex, BMI, ECOG-PS, TNM classification, histology type, tumor size, nuclear grade, tumor necrosis, preoperative serum CRP UISS, SSING, and COP-NLR. Each factor was assessed by contingency table with Chi-square analysis. A Kaplan-Meier analysis was carried out to estimate survival free ratio, and log-rank test was performed to compare the difference between assigned patient groups. On univariate and multivariate analysis, Cox proportional-hazard regression models, stratified by the factors described above, were used to estimate crude hazard ratios (HR) followed by calculating covariate-adjusted HR. All statistical tests were two sided, with P<0.05 considered to indicate statistical significance. All analyses were done using JMP® 12 (SAS Institute Inc., Cary, NC, USA).

## CONCLUSION

Increasing COP-NLR was associated with shorter patient survivals and an independent predictor for OS and RFS in localized RCC patients. As the COP-NLR can be measured preoperatively, this system should be incorporated in routine diagnosis for risk stratification and treatment decision-making of operable RCC patients.

## References

[1] Lipworth L, Tarone RE, McLaughlin JK (2011). Renal cell cancer among African Americans: an epidemiologic review. BMC Cancer.

[2] Chow WH, Dong LM, Devesa SS (2010). Epidemiology and risk factors for kidney cancer. Nat Rev Urol.

[3] Luo JH, Zhou FJ, Xie D, Zhang ZL, Liao B, Zhao HW, Dai YP, Chen LW, Chen W (2010). Analysis of long-term survival in patients with localized renal cell carcinoma: laparoscopic versus open radical nephrectomy. World J Urol.

[4] Adamy A, Chong KT, Chade D, Costaras J, Russo G, Kaag MG, Bernstein M, Motzer RJ, Russo P (2011). Clinical characteristics and outcomes of patients with recurrence 5 years after nephrectomy for localized renal cell carcinoma. J Urol.

[5] Komura K, Inamoto T, Black PC, Koyama K, Katsuoka Y, Watsuji T, Azuma H (2011). Prognostic significance of body mass index in Asian patients with localized renal cell carcinoma. Nutr Cancer.

[6] Tsujino T, Komura K, Matsunaga T, Yoshikawa Y, Takai T, Uchimoto T, Saito K, Tanda N, Oide R, Minami K, Uehara H, Jeong SH, Taniguchi K (2017). Preoperative measurement of the modified Glasgow prognostic score predicts patient survival in non-metastatic renal cell carcinoma prior to nephrectomy. Ann Surg Oncol.

[7] Crumley AB, McMillan DC, McKernan M, McDonald AC, Stuart RC (2006). Evaluation of an inflammation-based prognostic score in patients with inoperable gastro-oesophageal cancer. Br J Cancer.

[8] Pinato DJ, Shiner RJ, Seckl MJ, Stebbing J, Sharma R, Mauri FA (2014). Prognostic performance of inflammation-based prognostic indices in primary operable non-small cell lung cancer. Br J Cancer.

[9] Dutta S, Al-Mrabt NM, Fullarton GM, Horgan PG, McMillan DC (2011). A comparison of POSSUM and GPS models in the prediction of post-operative outcome in patients undergoing oesophago-gastric cancer resection. Ann Surg Oncol.

[10] Lindenmann J, Fink-Neuboeck N, Koesslbacher M, Pichler M, Stojakovic T, Roller RE, Maier A, Anegg U, Smolle J, Smolle-Juettner FM (2014). The influence of elevated levels of C-reactive protein and hypoalbuminemia on survival in patients with advanced inoperable esophageal cancer undergoing palliative treatment. J Surg Oncol.

[11] Hu H, Yao X, Xie X, Wu X, Zheng C, Xia W, Ma S (2017). Prognostic value of preoperative NLR, dNLR, PLR and CRP in surgical renal cell carcinoma patients. World J Urol.

[12] Ishizuka M, Nagata H, Takagi K, Iwasaki Y, Kubota K (2013). Combination of platelet count and neutrophil to lymphocyte ratio is a useful predictor of postoperative survival in patients with colorectal cancer. Br J Cancer.

[13] Ishizuka M, Oyama Y, Abe A, Kubota K (2014). Combination of platelet count and neutrophil to lymphocyte ratio is a useful predictor of postoperative survival in patients undergoing surgery for gastric cancer. J Surg Oncol.

[14] Feng JF, Huang Y, Chen QX (2014). The combination of platelet count and neutrophil lymphocyte ratio is a predictive factor in patients with esophageal squamous cell carcinoma. Transl Oncol.

[15] Zhang H, Zhang L, Zhu K, Shi B, Yin Y, Zhu J, Yue D, Zhang B, Wang C (2015). Prognostic significance of combination of preoperative platelet count and neutrophil-lymphocyte ratio (COP-NLR) in patients with non-small cell lung cancer: based on a large cohort study. PLoS One.

[16] Nakahira M, Sugasawa M, Matsumura S, Kuba K, Ohba S, Hayashi T, Minami K, Ebihara Y, Kogashiwa Y (2016). Prognostic role of the combination of platelet count and neutrophil-lymphocyte ratio in patients with hypopharyngeal squamous cell carcinoma. Eur Arch Otorhinolaryngol.

[17] General Assembly of the World Medical Association (2014). World Medical Association Declaration of Helsinki: ethical principles for medical research involving human subjects. J Am Coll Dent.

[18] Fuhrman SA, Lasky LC, Limas C (1982). Prognostic significance of morphologic parameters in renal cell carcinoma. Am J Surg Pathol.

[19] Zisman A, Pantuck AJ, Dorey F, Said JW, Shvarts O, Quintana D, Gitlitz BJ, deKernion JB, Figlin RA, Belldegrun AS (2001). Improved prognostication of renal cell carcinoma using an integrated staging system. J Clin Oncol.

[20] Frank I, Blute ML, Cheville JC, Lohse CM, Weaver AL, Zincke H (2002). An outcome prediction model for patients with clear cell renal cell carcinoma treated with radical nephrectomy based on tumor stage, size, grade and necrosis: the SSIGN score. J Urol.

[21] DeLong ER, DeLong DM, Clarke-Pearson DL (1988). Comparing the areas under two or more correlated receiver operating characteristic curves: a nonparametric approach. Biometrics.

[22] Gu L, Li H, Gao Y, Ma X, Chen L, Li X, Zhang Y, Fan Y, Zhang X (2015). The association of platelet count with clinicopathological significance and prognosis in renal cell carcinoma: a systematic review and meta-analysis. PLoS One.

[23] Proctor MJ, McMillan DC, Morrison DS, Fletcher CD, Horgan PG, Clarke SJ (2012). A derived neutrophil to lymphocyte ratio predicts survival in patients with cancer. Br J Cancer.

[24] Naito S, Yamamoto N, Takayama T, Muramoto M, Shinohara N, Nishiyama K, Takahashi A, Maruyama R, Saika T, Hoshi S, Nagao K, Yamamoto S, Sugimura I (2010). Prognosis of Japanese metastatic renal cell carcinoma patients in the cytokine era: a cooperative group report of 1463 patients. Eur Urol.

[25] Takahashi Y, Bucana CD, Akagi Y, Liu W, Cleary KR, Mai M, Ellis LM (1998). Significance of platelet-derived endothelial cell growth factor in the angiogenesis of human gastric cancer. Clin Cancer Res.

[26] Jefferson K, Persad R (2001). Poor prognosis associated with thrombocytosis in patients with renal cell carcinoma. BJU Int.

[27] Huang X, Wang L, Chen Y, Zheng X, Wang X (2017). Poor prognosis associated with high levels of thymidine phosphorylase and thrombocytosis in patients with renal cell carcinoma. Urol Int.

[28] George ML, Eccles SA, Tutton MG, Abulafi AM, Swift RI (2000). Correlation of plasma and serum vascular endothelial growth factor levels with platelet count in colorectal cancer: clinical evidence of platelet scavenging?. Clin Cancer Res.

[29] Gunsilius E, Petzer A, Stockhammer G, Nussbaumer W, Schumacher P, Clausen J, Gastl G (2000). Thrombocytes are the major source for soluble vascular endothelial growth factor in peripheral blood. Oncology.

[30] Sierko E, Wojtukiewicz MZ (2004). Platelets and angiogenesis in malignancy. Semin Thromb Hemost.

[31] Ramadori G, Van Damme J, Rieder H, Meyer zum Buschenfelde KH (1988). Interleukin 6, the third mediator of acute-phase reaction, modulates hepatic protein synthesis in human and mouse. Comparison with interleukin 1 beta and tumor necrosis factor-alpha. Eur J Immunol.

[32] Imai T, Koike K, Kubo T, Kikuchi T, Amano Y, Takagi M, Okumura N, Nakahata T (1991). Interleukin-6 supports human megakaryocytic proliferation and differentiation in vitro. Blood.

[33] Biffl WL, Moore EE, Moore FA, Barnett CC, Silliman CC, Peterson VM (1996). Interleukin-6 stimulates neutrophil production of platelet-activating factor. J Leukoc Biol.

[34] Mohle R, Green D, Moore MA, Nachman RL, Rafii S (1997). Constitutive production and thrombin-induced release of vascular endothelial growth factor by human megakaryocytes and platelets. Proc Natl Acad Sci U S A.

[35] Troxler M, Dickinson K, Homer-Vanniasinkam S (2007). Platelet function and antiplatelet therapy. Br J Surg.

[36] Symbas NP, Townsend MF, El-Galley R, Keane TE, Graham SD, Petros JA (2000). Poor prognosis associated with thrombocytosis in patients with renal cell carcinoma. BJU Int.

[37] Karam JA, Devine CE, Urbauer DL, Lozano M, Maity T, Ahrar K, Tamboli P, Tannir NM, Wood CG (2014). Phase 2 trial of neoadjuvant axitinib in patients with locally advanced nonmetastatic clear cell renal cell carcinoma. Eur Urol.

[38] Powles T, Sarwar N, Stockdale A, Sarker SJ, Boleti E, Protheroe A, Jones R, Chowdhury S, Peters J, Oades G, O’Brien T, Sullivan M, Aitchison M (2016). Safety and efficacy of pazopanib therapy prior to planned nephrectomy in metastatic clear cell renal cancer. JAMA Oncol.

[39] Rini BI, Plimack ER, Takagi T, Elson P, Wood LS, Dreicer R, Gilligan T, Garcia J, Zhang Z, Kaouk J, Krishnamurthi V, Stephenson AJ, Fergany A (2015). A phase II study of pazopanib in patients with localized renal cell carcinoma to optimize preservation of renal parenchyma. J Urol.

[40] Lane BR, Derweesh IH, Kim HL, O’Malley R, Klink J, Ercole CE, Palazzi KL, Thomas AA, Rini BI, Campbell SC (2015). Presurgical sunitinib reduces tumor size and may facilitate partial nephrectomy in patients with renal cell carcinoma. Urol Oncol.

[41] Chen L, Li H, Gu L, Ma X, Li X, Gao Y, Zhang Y, Shen D, Fan Y, Wang B, Bao X, Zhang X (2015). The impact of diabetes mellitus on renal cell carcinoma prognosis: a meta-analysis of cohort studies. Medicine (Baltimore).

[42] Kriegmair MC, Mandel P, Porubsky S, Durr J, Huck N, Nuhn P, Pfalzgraf D, Michel MS, Wagener N (2017). Metabolic syndrome negatively impacts the outcome of localized renal cell carcinoma. Horm Cancer.

[43] Sunela KL, Kataja MJ, Kellokumpu-Lehtinen PL (2013). Influence of body mass index and smoking on the long-term survival of patients with renal cell cancer. Clin Genitourin Cancer.

